# Transplanted Bone Marrow Mesenchymal Stem Cells Improve Memory in Rat Models of Alzheimer's Disease

**DOI:** 10.1155/2012/369417

**Published:** 2012-06-14

**Authors:** Parvin Babaei, Bahram Soltani Tehrani, Arsalan Alizadeh

**Affiliations:** ^1^Cellular and Molecular Research Center, Faculty of Medecine, Guilan University Complex, Rasht 41996-13769, Iran; ^2^Deptartment of Physiology, Faculty of Medecine, Guilan University Complex, Rasht 41996-13769, Iran; ^3^Deptartment of Pharmacology, Faculty of Medecine, Guilan University Complex, Rasht 41996-13769, Iran

## Abstract

The present study aims to evaluate the effect of bone marrow mesenchymal stem cells (MSCs) grafts on cognition deficit in chemically and age-induced Alzheimer's models of rats. In the first experiments aged animals (30 months) were tested in Morris water maze (MWM) and divided into two groups: impaired memory and unimpaired memory. Impaired groups were divided into two groups and cannulated bilaterally at the CA1 of the hippocampus for delivery of mesenchymal stem cells (500 × 10^3^/*μ*L) and PBS (phosphate buffer saline). In the second experiment, Ibotenic acid (Ibo) was injected bilaterally into the nucleus basalis magnocellularis (NBM) of young rats (3 months) and animals were tested in MWM. Then, animals with memory impairment received the following treatments: MSCs (500 × 10^3^/*μ*L) and PBS. Two months after the treatments, cognitive recovery was assessed by MWM in relearning paradigm in both experiments. Results showed that MSCs treatment significantly increased learning ability and memory in both age- and Ibo-induced memory impairment. Adult bone marrow mesenchymal stem cells show promise in treating cognitive decline associated with aging and NBM lesions.

## 1. Introduction

Alzheimer's disease (AD) has been called the disease of the century with significant clinical and socioeconomic impacts. Epidemiological studies point out that AD affects 5% of the population over 65 [1], and, parallel with increasing lifespan, the incidence of disease will rise dramatically. Clinically AD is characterized by a progressive learning capacity impairment and memory loss, especially memories of recent events [2–4]. One of the major pathological outcomes of both aging and Alzheimer's disease is loss of neurons and function in the basal forebrain [5–7] especially NBM, the main cholinergic input to the neocortex [8–10]. It is obvious that classical pathological hallmarks of AD are plaques and tangles, which both are exceptionally rare in animals, particularly in small laboratory rodents. In animal populations, as in humans, age-associated cognitive decline correlates with the degeneration of basal forebrain nuclei [1, 10]. Experimentally excitotoxic lesion of the NBM induces memory impairment in several tasks [11–13] and it is considered as a suitable approach to study cognitive deficit and dementia in animals [1, 12].

The current drug therapies for AD treatment are hindered due to poor efficacy and side effects [14, 15]. Adult neural tissues have limited sources of stem cells, which makes neurogenesis in the brain less likely. Stem cells transplantation seems to be a promising strategy for treatment of several central nervous system (CNS) degenerative diseases such as AD, amyotrophic lateral sclerosis (ALS), and Parkinson's disease [16, 17].

Bone marrow stem cells are an example of self-renewing multipotential cells with the developmental capacity to give rise to certain cell types [18, 19]. These cells seem to be able to differentiate into hepatocytes [20], skeletal muscle [21], cardiomyocytes [22], and neural cells [23–25] in vitro. Studies showed that implanted mesenchymal cells at the site of injury are able to survive and integrate in the host brain [1, 23, 26]. In this context Lee and coworkers [27] used human umbilical cord blood mesenchymal stem cells in AD mice and demonstrated cognitive rescue with restoration of learning and memory function. Also Nivet and coworkers [28] showed that human olfactory mesenchymal stem cells are able to restore learning and memory in hippocampus lesion model.

The ultimate goal for cell therapy in AD is functionality. Few studies have examined cognitive function with conflicting results: improvement [29, 30] and no change [31, 32]. Regarding the fact that using autologous cell transplantation circumvents ethical and immunological problems, the present study was aimed to evaluate the therapeutic effects of MSCs in restoring cognitive function in two different models of AD in rats.

## 2. Materials and Methods

### 2.1. Animals

All of the animals used in these experiments were housed in Cellular and Molecular Research Center animal facility. Animals were housed with free access to food and water in a 12 h light/dark cycle and constant temperature of 22°C. They were kept 4-5 in a cage. All procedures concerning animal care were in accordance with Guilan University of Medical Sciences Ethical Committee Article.

**Experiment 1**

Forty aged (30 months) and 10 young (3 months) male Wistar rats were used in this experiment. The mean weights were 500 ± 50 for old and 200 ± 20 g for young. Animals received four trials per day for 4 consecutive days in the Morris water maze (MWM) [33], using a 20 min intertrial interval. A probe trial during which the platform was removed was carried out on the fifth day. Rats above the mean average of latency designated as impaired were divided into grafted (*n* = 10) and nongrafted control groups (*n* = 10). Animals were placed in a computerized stereotaxic apparatus (Neurostar, Germany) and cannulated at CA1 region (at coordinates AP: −3 mm, L: ±2 mm from bregma and V: −2.8 mm from the skull surface) [34]. Performance of aged grafted animals was compared with aged nongrafted and young control groups.

**Experiment 2 (NBM Lesion)**

Forty male Wistar rats (3 months old, weighing 200 ± 20 g) were used in this part of study. To establish cognitive deficit, we infused Ibo into the NBM. On the day of surgery, the animals were anesthetized with ketamine/xylazine (50 mg/kg, i.p.) and placed in stereotaxic apparatus. The incisor bar was set at −1.14 mm posterior and ±2.46 mm lateral to the bregma and 7.9 below the top of the skull to reach the nucleus basalis magnocellularis [12], then guide cannula was implanted bilaterally for further infusions. Another cannula for stem cell transfusion was implanted in the CA1 at coordinates mentioned in Experiment 1. Rats received bilateral infusions of 0.5 *μ*L of Ibo (10 *μ*g/*μ*L) using a 5 *μ*L Hamilton syringe. After 14 days, rats were tested in MWM in order to test learning ability. Animals that showed memory impairment were distributed into two groups: Ibo + MSCs (*n* = 10) and Ibo + PBS (*n* = 10).

### 2.2. Bone Marrow Stem Cells Isolation

Rat bone marrow was obtained by aspiration from tibia. This study was approved by the Institutional Ethical Committee of Guilan University of Medical Sciences. Bone marrow was collected and centrifuged with ficoll for 10 min at 1500 xg; the white blood cells buffy coat was recovered and plated in 75 cm flasks containing with Dulbecco's Modified Eagle's Medium (DMEM) and 10% fetal bovine serum (FBS). Cells were then incubated at 37°C in humidified atmosphere containing 95% air and 5% CO_2_. On reaching confluence, the adherent cells were detached by 0.05% trypsin and 0.02% ethylenediaminetetraacetic acid (EDTA) for 5–10 min at 37°C, harvested and washed with DMEM, and resuspended in medium containing 10% FBS. After the first passage, the morphologically homogeneous population of MSC was analyzed for the expression of cell surface molecules using flow cytometry procedures for CD105, CD90, and CD44+. The ability of MSCs to differentiate to adipogenic lineages was assayed using adipogenic media (acid ascorbic 50 *μ*g/mL, dexametazon 100 nM, indometacin 5 *μ*g/mL, and insulin 5 *μ*g/mL). Viability of cells was determined by Trypan blue dye exclusion test. Briefly, cells were incubated with Trypan blue dye for 1 min. Blue positive and white negative cells was counted in ten 20× fields, and the percent of viable cells was calculated.

 Both grafted groups received infusion of 1 *μ*L (500 × 10^3^/*μ*L) cells from passage 2, and controls received the same volume of PBS into the CA1 of the hippocampus. The syringe was allowed to remain in place for 5 min after the injection to allow diffusion into the surrounding tissue.

### 2.3. Behavioral Tests

Two months after transplantation, rats performed relearning task (the place of platform was different from the previous experiment) in Morris water maze. The Morris water maze consisted of a black pool (148 cm diameter) filled with water (26 ± 2°C). A circular black platform was submerged 2 cm below the water surface, in the middle of the target quadrant. The behavior of the rats in the pool could be tracked with a camera connected to Ethovision system (Ethovision XT 7, Noldus inc., The Netherlands) allowing us to measure swim speed, distance, and latency to find the platform. Rats were trained with a protocol of four trials per day, with an interval of 20 min, for 4 consecutive days. A probe trial was administered on the fifth day, when each subject was placed into the water diagonally opposite the target quadrant and allowed 90 seconds to search the water, from which the platform had been removed.

### 2.4. Statistical Analysis

The data is expressed as means ± SEM. Group differences in the escape latency of probe task in the Morris water maze were analyzed using one-way analysis of variance (ANOVA) followed by Tukey's post hoc test. ANOVA repeated measure for multiple group comparison was used to analyze group differences of the data collected during the training days.

## 3. Results

### 3.1. Stem Cells Characterization

**Figure 1:**
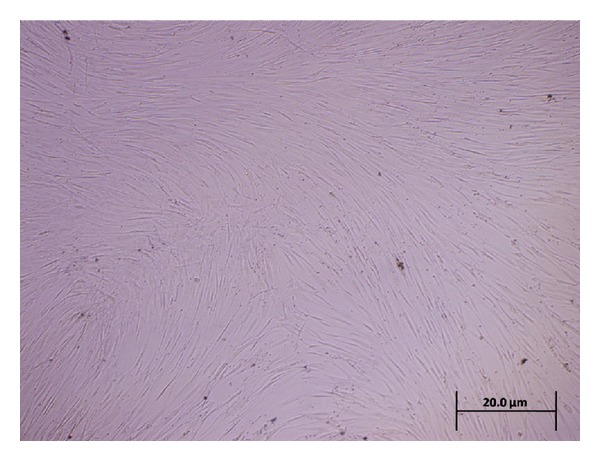
Inverted microscope photomicrograph shows morphological characteristic of MSCs (spindle shape) derived from rat bone marrow in passage 3. Scale bar: 20 *μ*m.

**Figure 2:**
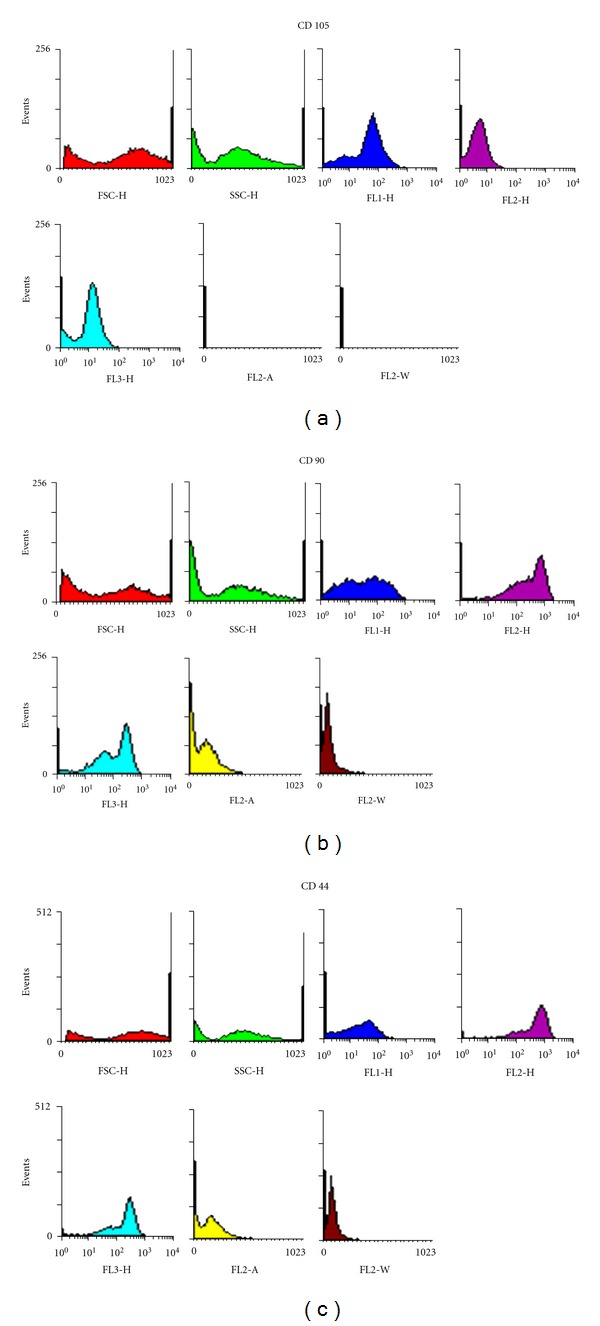
Flow cytometry analysis of CD 105, CD 90, and CD 44 in rat MSCs. Results represent three independent experiments.

Mesenchymal stem cells were successfully cultured and expanded. A morphologically homogeneous population of fibroblast-like cells (Figure 1) with more than 90% confluence was seen after 14 days. Cells after the first passage grew exponentially, requiring weekly passages. Flow cytometric analysis was used to assess the purity of MSC cultures, which appeared uniformly positive for CD44, CD105, and CD90 (Figure 2).

## 4. Behavioral Tests

### 4.1. Age-Induced Memory Impairment

**Figure 3:**
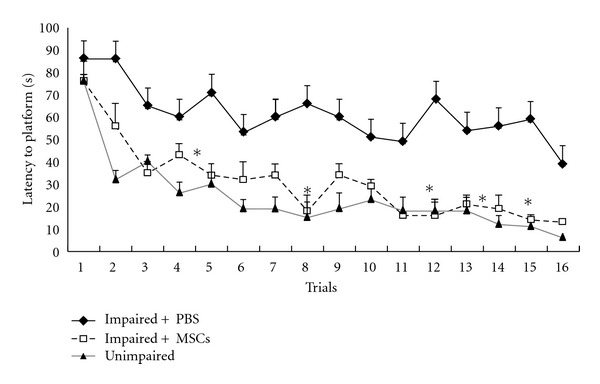
Comparisons of the acquisition performance on the Morris water maze task among the three groups. The results are the mean swimming time traveled per trial toward the platform. The mean values of the 16 trials for 4 days for each group are shown. Repeated measures of ANOVA for the swimming time among the groups were followed by Tukey's test. *P < 0.05 and **P < 0.01 as compared with the corresponding data from the impaired +PBS group. Performance was assessed two months after the treatments.

During the training sessions in the MWM, unimpaired, impaired + PBS, and impaired + MSCs groups showed significant trial effects in learning procedure (*F*_2,  445_ = 5.138,  *P* < 0.0001) (Figure 3). Since none of the groups differed in swimming speed (22.3 ± 0.8 versus 23 ± 1.9 cm/s; *P* > 0.05), the latency to find platform was used as an indicator of learning performance. There was no interaction between the trials and the groups (*F*_2,  445_ = 1.273, *P* = 0.273). Impaired + MSCs rats learned to find the platform more rapidly than impaired + PBS (*F*_2,  25_ = 36.799, *P* < 0.001, *n* = 9, Figure 3). One rat from the cell transplanted group died after one month due to brain infection.

**Figure 4:**
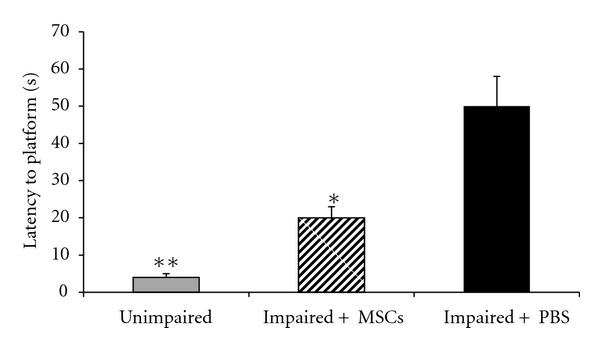
Comparisons of the retention performance on the Morris water maze task among the three groups two months after the treatments (unimapaired, impaired + PBS, impaired + MSCs). The mean values of the probe test for each group are shown. One-way ANOVA for the swimming time among the groups was followed by Tukey's test. *P < 0.05 as compared with the corresponding data from the impaired + PBS group.

There was significant difference in probe latency between impaired + MSCs and impaired + PBS animals (11.5 ± 0.88 versus 33.4 ± 8.48 s, *P* = 0.006, Figures 4 and 7). Although the impaired + MSCs group showed improvement in latency to target quadrant, they did not reach the young group score (11.5 ± 0.88 versus 4 ± 0.45 s).

### 4.2. Ibo-Induced Memory Impairment

**Figure 5:**
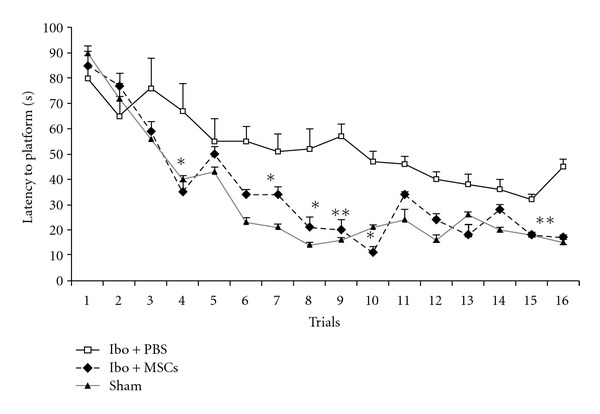
Comparisons of the acquisition performance on the Morris water maze task among the three groups of the Ibo-lesioned rats. The results are the mean latency time traveled per trial. The mean values of the 16 trials for 4 days for each group are shown. Repeated measures of ANOVA for the swimming time among the groups. *P < 0.05 and **P < 0.01 as compared with the corresponding data from the Ibo + PBS group.

**Figure 6:**
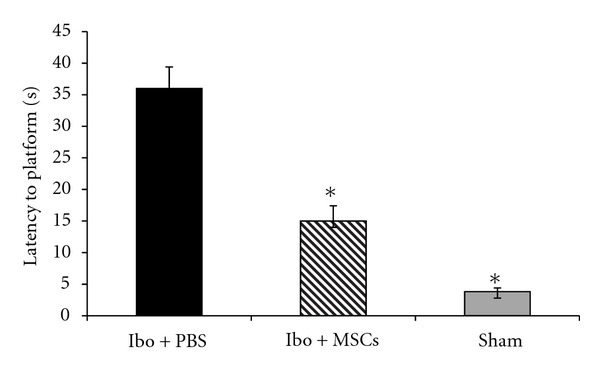
Comparisons of the retention performance on the Morris water maze task among the three groups of the rats. The results are the mean percentage of latency time to the platform in the probe test. The mean values of the four trials for each group are shown. Mean of swimming time among the groups was analyzed using one-way ANOVA and post hoc Tukey's test. *P < 0.05 and **P < 0.01 as compared with the corresponding data of the Ibo + PBS group.

**Figure 7:**
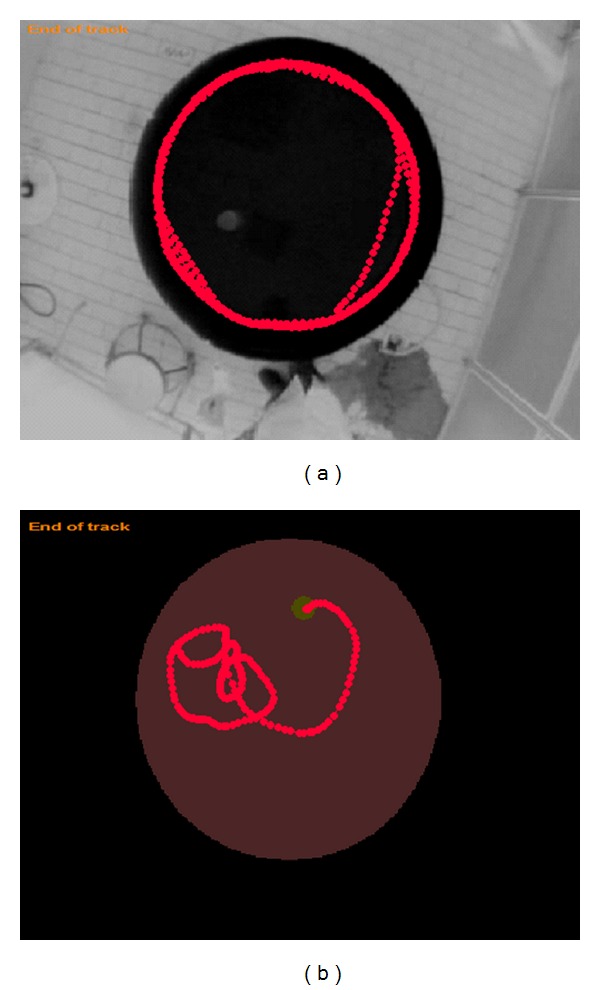
Example of computer tracking from probe trial (90 s duration). (a): “aged-impaired + PBS”; (b): “aged-impaired + MSCs”. The rat of “aged-impaired” swims in a concentric pattern.

Acquisition of the Morris water maze task in Ibo-lesioned groups is demonstrated in Figure 5. During the experiment, the latency to escape diminished over time in lesioned and sham operated groups (*F*_2,445_ = 26.310,  *P* < 0.001). There was no interaction between the group and the trials (*F*_2,445_ = 1.349,  *P* = 0.212). Ibotenic acid severely impaired the latency to platform in the probe test compared to sham group (37 ± 1.5 versus 3.8 ± 0.6 s *P* < 0.0001). Ibotenic acid had no significant effect on speed of swimming (20 ± 0.82 versus 21.8 ± 1.5 cm/s). Two months after grafting the MSCs, rats learned to find the platform quickly. As expected, the rats showed less time needed to find the platform (*F* = 64.689, *P* < 0.0001). Tukey's post hoc test showed that the Ibo + MSCs significantly reduced the latency to find the platform compared with Ibo + PBS group (14 ± 2.4 versus,  34 ± 3.4  s, Figure 6). Total time spent in the target quadrant also significantly increased in Ibo + MSCs compared with Ibo + PBS (28.6 ± 2.4 versus 12.8 ± 2.08 s, *P* < 0.0001).

The results showed that stem cell treatment attenuated Ibo-induced learning and memory impairment in the Morris water maze test.

## 5. Discussion

 The purpose of this study was to evaluate the therapeutic effects of transplanting MSCs in memory impairment induced by aging and excitotoxic lesion of NBM. The aged animals used in our experiment showed sever impairment in spatial learning, attention, and memory. According to previous findings, cognition deficit in these animals correlates with the degenerative decline of basal forebrain nuclei [8, 9]. It seems that using aged animals is appropriate to evaluate memory function.

 In the second part of our study, the infusion of Ibo into the NBM produced significant disruption of the working memory, which is in agreement with other studies indicating association of this nucleus with working memory [35–37]. Cholinergic neurons of NBM projecting to the hippocampus play major role in cognitive performance such as attention, learning, and memory. It has been shown that infusion of Ibo decreases cholinergic activities in the hippocampus and frontal cortex [38] via hyperstimulation of the N-methyl-D-aspartate receptor [39, 40].

 Our data from both animal models shows that there is a significant improvement in learning and memory following MSCs transplantation. These results confirm the ultimate objective of stem cells transplantation, which is achievement of cognitive functional recovery. Since Ibo leads to specific loss of somata of various neuron types without affecting on other surrounding cells, such as glia and endothelial cells or even neural axons [39, 40], our data indicates that transplanted stem cells probably differentiated to neurons in hippocampus. It has been known that this area is a very sensitive region of the brain that plays a pivotal role in encoding, consolidating, and retrieving learning and memory [41]. Improvement of learning and memory in our study is in agreement with previous studies using other sources of stem cells including neural [42–44], olfactory [28], and umbilical cord blood stem cells [27]. Nivet et al. [28] indicated that transplanted olfactory MSCs not only stimulate endogenous neurogenesis but also restore synaptic transmission and enhance long-term potentiation. A study conducted by Lee et al. [27] demonstrated that human umbilical cord blood mesenchymal stem cells transplantation reduces glial activation, oxidative stress, and apoptosis in AD mouse brain and consequently improves memory and learning.

 Although the present study does not aim to study the mechanisms underlying memory improvement, several mechanisms could possibly contribute to the improvement in learning and memory after stem cell transplantation in our experiments. One is the capability of these cells to add to the pool of functioning neurons [24, 45–48] and integrating with neighboring cells [1, 23, 26]. This mechanism needs to be supported in the future studies by electrophysiological integration of the stem cells into the host circuitry. We initiated behavioral tests two months after transplantation, which provides enough time for mesenchymal stem cells to develop synapses and electrophysiological response based on observations in previous studies in other neurodegenerative diseases and in vitro studies [45, 48]. Second possibility is that stem cells may provide therapeutic utility by enhancing the survival and activity of the existing neurons [46, 49]. Wu et al. [17] in their review article stated that neural stem cells release diffusible factors that may improve the survival of aged and degenerating neurons in human brains [17].

 Mesenchymal stem cells are very attractive in view of a possible cell therapy approach in neurodegenerative diseases because of their great plasticity. Recently, MSCs therapy has been shifted to be used in some clinical trial models like ALS [48, 49]. A phase I clinical trial conducted by Mazzini confirmed that MSCs transplantation into the spinal cord of ALS patients is safe and that MSCs might have a clinical use for future ALS cell-based clinical trials [48].

In conclusion, MSC grafts reverse progressive cognitive decline associated with aging and Ibo lesion in animal models.

 From a clinical point of view, considering low risk of tumourigenesis [48, 50, 51] and less ethical issues with bone marrow mesenchymal stem cells, these cells represent as a valuable candidate source for transplantation therapy in Alzheimer's disease. 

## Supplementary Material

Figure 8. Comparisons of the retention performance on the Morris water maze task among the three groups of the rats. The results are the mean percentage of total time spent in the target quadrant in the probe test. Mean of swimming time among the groups were analyzed using one-way ANOVA and post hoc Tukey's test. ∗P< 0.05, as compared with the corresponding data of the impaired+PBS group.
Figure 9. Comparisons of the retention performance on the Morris water maze task among the three groups of the rats. The results are the mean percentage of total time spent in the target quadrant in the probe test. Mean of swimming time among the groups were analyzed using one-way ANOVA and post hoc Tukey's test. ∗P< 0.05, as compared with the corresponding data of the Ibo+PBS group.
Figure 10. NBM cannulated.
Figure 11. Adipocytes differentiated from MSCs after treatment in adipogenic medium. Adipose vacuoles staining pink color due to red oil represent differentiation toadipocyte. magnification 40X.
