# Supplementary material for: Transplanted Bone Marrow Mesenchymal Stem Cells Improve Memory in Rat Models of Alzheimer's Disease
Source: Stem Cells Int. 2012 Jun 14;2012:369417. doi: 10.1155/2012/369417 (PMC3382392; doi:10.1155/2012/369417)
Supplement: Supplementary file 1 — Figure 8. Comparisons of the retention performance on the Morris water maze task among the three groups of the rats. The results are the mean percentage of total time spent in the target quadrant in the probe test. Mean of swimming time among the groups were analyzed using one-way ANOVA and post hoc Tukey's test. ∗P< 0.05, as compared with the corresponding data of the impaired+PBS group. Figure 9. Comparisons of the retention performance on the Morris water maze task among the three groups of the rats. The results are the mean percentage of total time spent in the target quadrant in the probe test. Mean of swimming time among the groups were analyzed using one-way ANOVA and post hoc Tukey's test. ∗P< 0.05, as compared with the corresponding data of the Ibo+PBS group. Figure 10. NBM cannulated. Figure 11. Adipocytes differentiated from MSCs after treatment in adipogenic medium. Adipose vacuoles staining pink color due to red oil represent differentiation toadipocyte. magnification 40X. [file 369417.f1.docx]

**Fig 8, 9 ,10, 11 as supplementary**

**Fig 8** . Comparisons of the retention performance on the Morris water maze task among the three groups of the **rat**s. The results are the mean percentage of of total time spent in the target quadrant in the probe test. .Mean of swimming time among the groups were analyzed using one-way ANOVA and post hoc Tukey's test. **P*< 0.05, as compared with the corresponding data of the impaired+PBS group

**Fig 9**

. . Comparisons of the retention performance on the Morris water maze task among the three groups of the **rat**s. The results are the mean percentage of of total time spent in the target quadrant in the probe test. .Mean of swimming time among the groups were analyzed using one-way ANOVA and post hoc Tukey's test. **P*< 0.05, as compared with the corresponding data of the Ibo+PBS group

**Fig 10 : NBM cannulated**


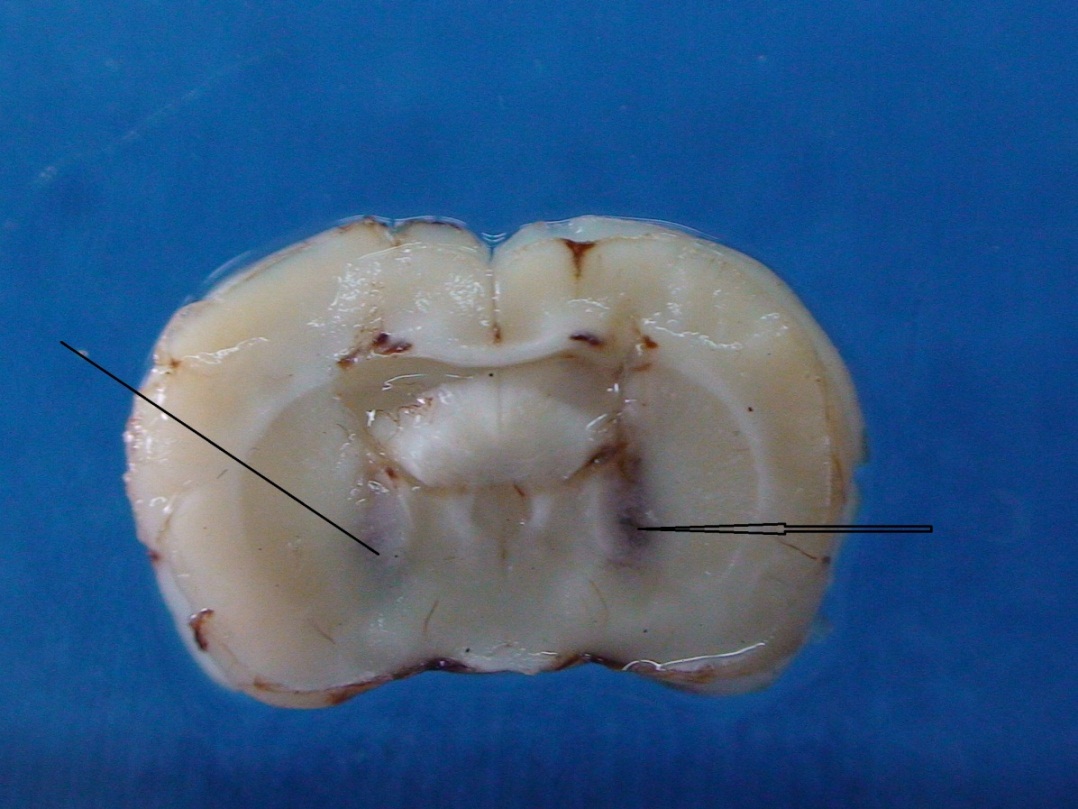


**Fig 11 differentiation to Adipocytes**


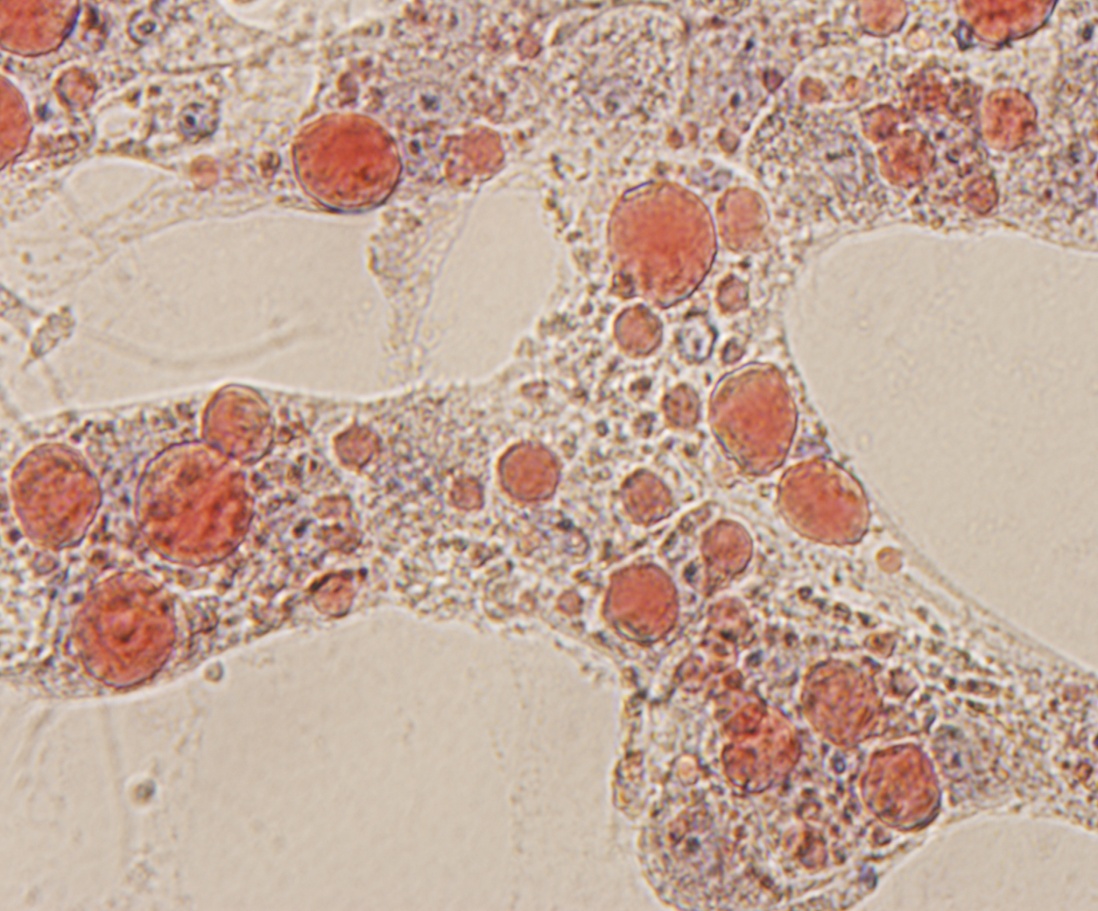


**adipocytes differentiated from MSCs after treatment in adipogenic medium. Adipose vacuoles staining pink color due to red oil represent differentiation toadipocyte . magnification 40X**
